# Non-absorbable antibiotic treatment inhibits colorectal cancer liver metastasis by modulating deoxycholic acid metabolism by intestinal microbes

**DOI:** 10.7150/jca.63490

**Published:** 2022-01-01

**Authors:** Junjie Deng, Wei Yuan, Qin Tan, Xundong Wei, Jie Ma

**Affiliations:** 1 State Key Laboratory of Molecular Oncology, National Cancer Center/National Clinical Research Center for Cancer/Cancer Hospital, Chinese Academy of Medical Sciences & Peking Union Medical College, Beijing 100021, P.R. China.; 2 Center of Biotherapy, Beijing Hospital, National Center of Gerontology; Institute of Geriatric Medicine, Chinese Academy of Medical Sciences & Peking Union Medical College, Beijing, 100730, P.R. China.

**Keywords:** Microbes, Colorectal cancer, Tumor metastasis, Deoxycholic acid

## Abstract

Emerging evidence suggests that intestinal microbes influence the occurrence and development of colorectal cancer (CRC). However, few studies have examined the relationship between gut bacteria and liver metastasis of CRC. In this study, we found that administration of non-absorbable antibiotics inhibited liver metastasis of CRC in a mouse model compared with a control group. To elucidate the underlying mechanism, immune cell infiltration analysis, 16S rRNA sequencing, and metabolomics were performed. Differential analysis revealed that non-absorbable antibiotic treatment significantly altered gut microbial diversity and decreased the concentration of deoxycholic acid (DCA) in feces and liver tissues. Furthermore, we verified that bacteria capable of converting cholic acid (CA) to DCA via 7α-dehydroxylation were reduced in mice treated with non-absorbable antibiotics. Finally, *in vitro* and *in vivo* experiments confirmed that DCA accelerated the proliferation and metastasis of CRC cells.

## Introduction

Colorectal cancer is one of the most common digestive tract cancers and ranks third in morbidity and mortality worldwide 1. Common clinical treatments for CRC include surgery, chemotherapy, radiotherapy and immunotherapy 2, 3, but these methods have limited effectiveness for advanced CRC. Metastatic CRC remains the main cause of death in CRC patients, and the quality of life of patients with advanced CRC is poor 4.

CRC is generally thought to arise from gene mutation and the interaction between cancer cells and the environment 5. In particular, many recent studies have reported a relationship between gut microbes and CRC development 6, 7. Gut microbes may regulate the occurrence and development of cancer in three ways: direct toxic effects, regulation of the immune system, and regulation of metabolic products 8. Among metabolic products, the bile acids pathway has been shown to play an important role in carcinogenesis 9. Primary bile acids are synthesized through cholesterol transfer in the liver and then secreted into the intestines for lipid digestion 10. Ninety percent of primary bile acids are reabsorbed in the ileum, and the remaining 10% flow to the colon and are dehydroxylated by gut microbes to become secondary bile acids 11. The liver and colon are connected through the portal vein, and thus microbes and their metabolites can be directly transferred from the intestine to the liver and influence the liver microenvironment. Deoxycholic acid (DCA), a secondary bile acid formed by the metabolism of the primary bile acid cholic acid (CA) by intestinal microbes, has been implicated in colorectal and liver cancer development 12, 13.

Although the relationship between intestinal microbes and tumor formation has been widely investigated, the effects of these microbes on cancer metastasis have received little attention 14, 15. Previous studies have used antibiotic cocktails (ABX) to alter the diversity of intestinal microbes, but these cocktails are absorbable through the gut and may affect distal organs 14. As a result, it is difficult to differentiate the direct effects of these antibiotics in organs from the effects of changes in microbial composition 16. Therefore, it is particularly important to study the direct relationship between the intestinal microbiota and cancer metastasis and elucidate the underlying mechanisms.

In this study, we found that treatment with non-absorbable antibiotics alters the composition of gut microbes and inhibits liver metastasis of CRC. Integrated analysis of immune cells, 16S rRNA sequencing, and metabolomics demonstrated that DCA is a key metabolite in our antibiotic treatment model that can promote cancer proliferation and metastasis *in vitro* and *in vivo*.

## Materials and methods

### Mice and mouse models

BALB/c and C57BL/6J mice (six to eight weeks of age) were purchased from Beijing HFK Bioscience Co. Ltd. All animals were maintained under specific pathogen-free (SPF) conditions. For non-absorbable antibiotic treatment, mice were orally administered 100 μl of gentamicin (5 mg/ml) and amikacin (5 mg/ml, Yichang Humanwell Pharmaceutical Co. Ltd) daily for two weeks. Mouse models of tumor formation and liver metastasis were constructed by spleen injection of 1.5×10^6^ CT26luc tumor cells or 2×10^6^ MC38 tumor cells. The mouse model of lung metastasis was constructed by injection of 3×10^5^ B16 tumor cells via the tail vein. To assess the effect of DCA on cancer metastasis, mice were orally given 40 μg/g DCA (Sigma, USA) daily according to previous study and followed with spleen injection of tumor cells 17. All animal experiments followed protocols approved by the Animal Care and Use Committee of National Cancer Center (Beijing, China).

### Patients and specimens

This research was approved by the ethical committee of the Beijing Hospital (Beijing, China, ethical number: 2018BJYYEC-106-01). Blood samples were collected from 8 patients with metastatic cancer and 10 patients without metastatic cancer. Plasma was extracted from blood by centrifugation for further DCA detection and analysis.

### Antibodies and flow cytometry

Antibodies against CD16/CD32, CD45(I3/2.3), CD4(RM4-5), CD8a(53-6.7), CD49b(DX5), CD25(CD25-4E3), FOXP3(FJK-16s), CD19(1D3), CD11b(1CRF44), Ly6C(HK1.4), Ly6G(RB6-8C5), F4/80(BM8), CD3e(145-2C11), and CD27(LG.7F9) were purchased from eBioscience (California, USA). Flow cytometry was performed as described previously 18.

### Measurement of serum alanine aminotransferase (ALT) and lipopolysaccharide (LPS)

The concentration of serum ALT was determined using the biochemistry kit from Solarbio Life Science (Beijing, China) according to the manufacturer's instructions. The concentration of serum LPS was determined by using an ELISA kit from MEIMIAN Co. Ltd (Jiangsu, China).

### *In vivo* imaging of mice

Twenty-five minutes before beginning photon recording, the mice were intraperitoneally injected with 200 μl of D-luciferin sodium salt (15 mg/ml). Next, mice were euthanasia and removed the liver to place in a dark chamber, and the position of the liver to be imaged was confirmed under dim light. Then, a cooled charged-coupled device (CCD) camera was utilized to detect the fluorescence intensity.

### Bacterial 16S rRNA sequencing and analysis

Microbial community genomic DNA was extracted from 50 mg of feces or 50 mg of lung tissue using a DNA extraction kit (Omega, USA) according to the manufacturer's instructions. The universal primer pair 338F (5'-ACTCCTACGGGAGGCAGCAG-3') and 806R (5'-GGACTACHVGGGTWTCTAAT-3') was used to amplify the V3-V4 hypervariable region of the microbial 16S rRNA gene. Purified amplicons were sequenced, and data were pre-processed according to the standard protocols of Majorbio Biology (Shanghai, China). The PICRUST package was used to predict functional changes based on the 16S rRNA sequencing results as described previously 19.

### Liquid chromatography-mass spectrometry (LC-MS) metabolomics and RNA sequencing

Fresh mouse liver tissue and fecal samples (50 mg) were used for LC-MS non-targeted metabolomics and RNA sequencing according to the standard protocols of Majorbio Biology (Shanghai, China). Differential metabolites were identified based on a variable importance of projection (VIP) > 1 and student's *P*-value < 0.05. To analyze changes in bile acids, LC-MS-based targeted metabolomics was performed. The bile acid metabolites in 150 μl of serum from mouse or plasma from clinical patients were quantified according to the standard protocols of Majorbio Biology (Shanghai, China). Extreme values were eliminated by Grubbs' test with the threshold value of 0.95. Metabolites with relative standard deviations (RSD) < 15% were considered for further analysis.

### Bacterial quantitative PCR (QPCR)

QPCR was performed with DNA extracted from 150 mg of feces using the following primers: gram-negative bacteria, 5'-CATCGTTTACGGCGTGGAC-3' (forward) and 5'-AGAGATATGGAGGAACACCA-3' (reverse); bacterial *BaiJ* 17, 5'-TCAGGACGTGGAGGCGATCCA-3' (forward) and 5'-TACRTGATACTGGTAGCTCCA-3' (reverse). QPCR amplification of DNA was performed as follows: initial denaturation at 95 °C for 3 min; 40 cycles of 95 °C for 5 s and 60 °C for 34 s; and melting curve detection.

### Cell culture and cell counting kit-8 assay

Mouse CT26luc cells were purchased from Biohelix Biotech Co. Ltd (Guangzhou, China), and mouse MC38 and B16-F10 cells were prepared from stocks in our laboratory. Cells were cultured in RPMI medium (Cytiva, USA) with 10% fetal bovine serum (FBS, Gibco, USA) in 5% CO_2_. Cell proliferation was assessed via cell counting kit-8 (CCK8; Dojindo, Japan) as follows. First, 1×10^5^ cells were inoculated into a 96-well plate and incubated at 37 °C and 5% CO_2_ for 24 h. Next, a gradient of concentrations of DCA was added to the medium and incubated for 24 h. Finally, 10 μl of CCK8 reagent was added to the medium, and the absorbance at 450 nm was measured.

### Transwell migration assay

Transwell migration assays were performed in a Transwell chamber (Costar, USA). The upper chamber contained 200 μl of serum-free medium inoculated with 7×10^5^ cells, and the low chamber was filled with 600 μl of medium containing 10% FBS. After incubation for 24 h, the cells in the bottom chamber were fixed in 4% paraformaldehyde solution (Solarbio, China) for 30 min and stained with Giemsa's stain (Solarbio, China) for 30 min.

### Statistical analysis

Results are presented as the mean ± SD. R software (version 4.0.2, www.R-project.org) was used for Student's t-test analysis, and *P* < 0.05 was considered statistically significant. Anosim analysis was used to obtain *P-*values in principal component analysis (PCA). Figures were plotted using GraphPad Prism 5.0 (GraphPad Software, www.graphpad.com) and the ggplot2 package of R software 20.

## Results

### Non-absorbable antibiotic treatment inhibits tumor liver metastasis in mouse models

**Figure 1:**
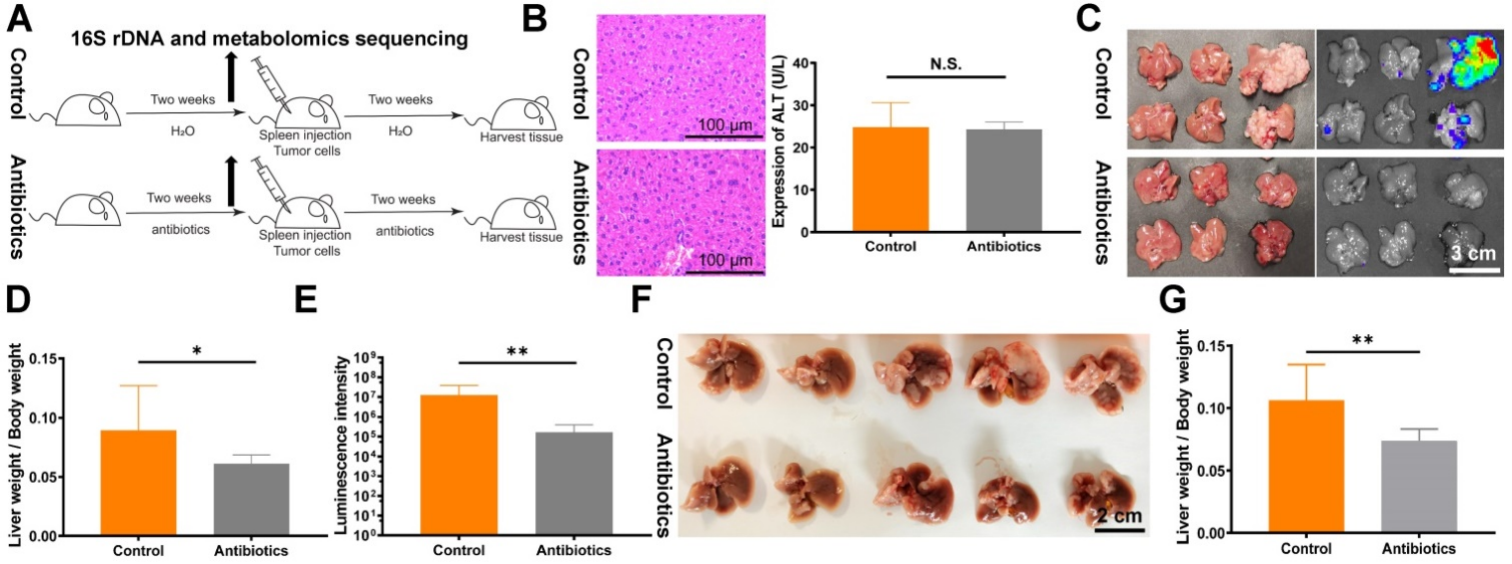
** Non-absorbable antibiotic treatment inhibits tumor liver metastasis in mouse models. (A)** Flowchart of the antibiotic treatment model. **(B)** HE staining of mouse liver tissues and analysis of mouse blood ALT levels revealed no significant changes after two weeks of antibiotic treatment. Sections were observed under a microscope at 400×. **(C)** Liver metastasis in BALB/c mice after spleen injection of 1.5×10^6^ CT26luc cells. **(D)** Metastasis indexes and **(E)** luminescence intensities showed that antibiotic treatment significantly reduced liver metastasis in BALB/c mice. **(F)** Liver metastasis in C57BL/6J mice after spleen injection of 2×10^6^ MC38 cells. **(G)** Metastasis indexes showed that antibiotic treatment significantly decreased liver metastasis in C57BL/6J mice. N.S., no significance; *P < 0.05, **P < 0.01.

To directly target the intestinal microbial composition in mice, we used gentamicin and amikacin, which are non-absorbable antibiotics (Fig. 1A). No significant effects of non-absorbable antibiotics on ALT expression and HE staining were observed (Fig. 1B). Next, we verified the impact of changes in intestinal microbes on tumor proliferation and metastasis. A mouse liver metastasis model was constructed by spleen injection of CT26luc cells. Compared with the control group, the liver metastasis index and *in vivo* imaging fluorescence intensity were lower in the antibiotic treatment group (Fig. 1C-E). The decrease in liver metastasis due to antibiotic administration was further verified in the C57BL/6 mouse spleen injection liver metastasis model (Fig. 1F and 1G). Finally, we assessed the effects of antibiotic treatment on tumor metastasis and formation in other mouse models. However, we did not observe significant effects on tumor metastasis and formation in the tail vein injection lung metastasis model (Fig. S1A and S1B) and the subcutaneous flank tumor model (Fig. S1C).

### Non-absorbable antibiotic treatment alters the composition of the gut microbiota

**Figure 2:**
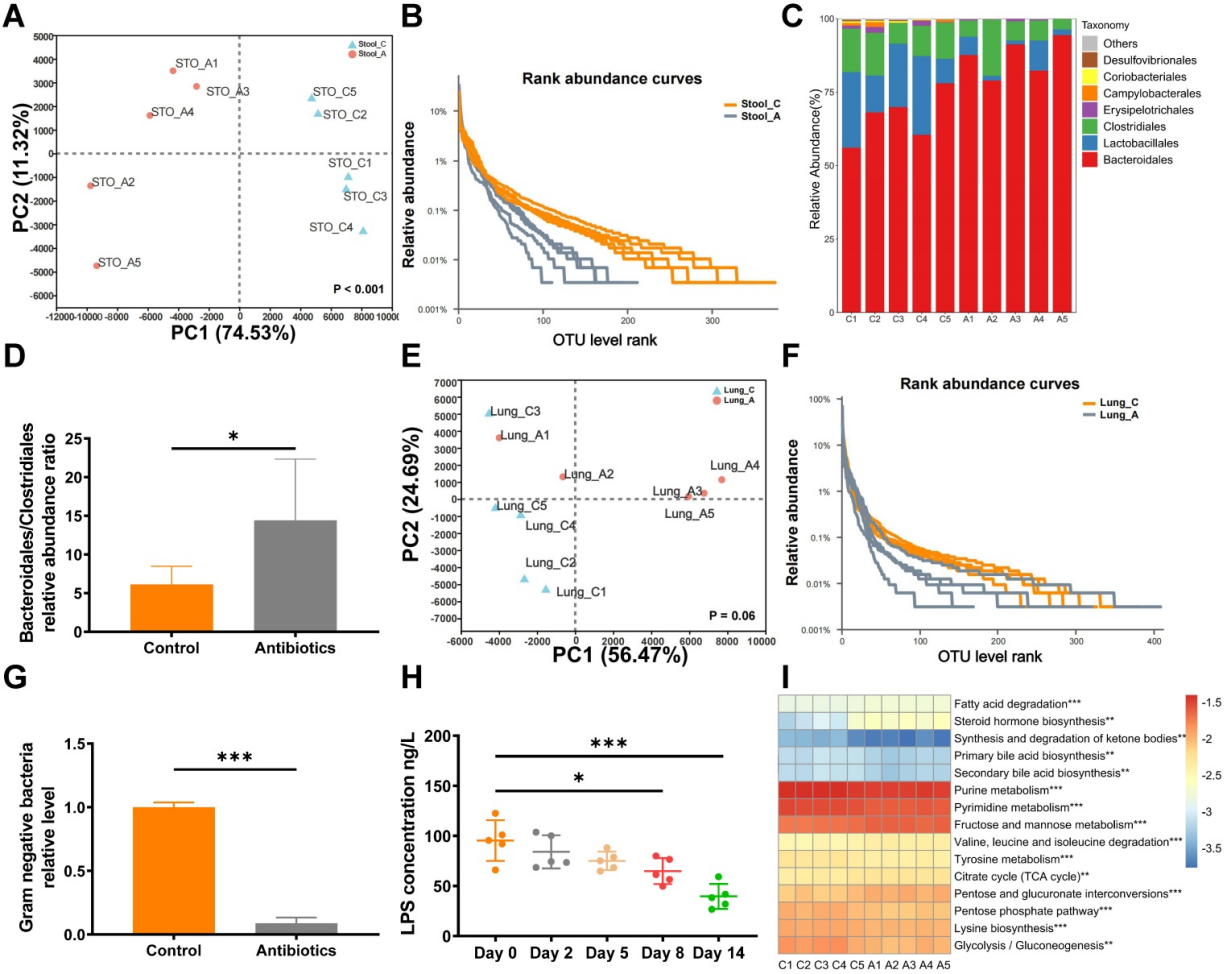
** Non-absorbable antibiotic treatment alters the composition of the gut microbiota. (A)** PCA showed that OTU diversity changed significantly after antibiotic treatment. Anosim analysis was used to obtain *P-*values. **(B)** Rarefaction curves of microbes at the OTU level. **(C)** Bar plot of microbial diversity at the order level. **(D)** Ratio of the relative abundances of Bacteroidales to Clostridiales. **(E)** PCA analysis and **(F)** rarefaction curve of microbes in lung tissues at the OTU level after antibiotic treatment. **(G)** Quantitative PCR showed that two weeks of antibiotic treatment reduced the levels of gram-negative bacteria. **(H)** ELISA demonstrated that blood LPS levels were reduced after antibiotic treatment. **(I)** Heatmap of top 15 altered KEGG pathways according to PICRUST analysis. *P < 0.05, **P < 0.01, *** P < 0.001.

To assess microbial changes after antibiotic gavage, the gut and lung microbiota were analyzed by 16S rRNA sequencing after two weeks of treatment with non-absorbable antibiotics. PCA revealed that non-absorbable antibiotic treatment significantly changed the diversity of operational taxonomic units (OTUs) in the gut microbiota (Fig. 2A). Rank abundance curves revealed that OTU diversity was lower in the antibiotic treatment group than the control group (Fig. 2B). Moreover, the relative abundance ratio of Bacteroidales to Clostridiales was significantly higher in the antibiotic treatment group (Fig. 2C and 2D) and was negatively correlated with microbial diversity at the order level 21. Thus, antibiotic treatment greatly reduced intestinal microbial diversity. However, changes in diversity were not apparent in 16S rRNA sequencing analysis of lung tissue (Fig. 2E and 2F), which may be attributed to the use of non-absorbable antibiotics.

In clinical application, aminoglycosides have been shown to significantly decrease gram-negative bacteria. QPCR analysis revealed that treatment with non-absorbable antibiotics effectively reduced levels of gram-negative bacteria in fecal samples (Fig. 2G). As further verification, concentrations of LPS in mouse blood were analyzed by ELISA. Interestingly, we observed that the blood concentration of LPS decreased over time and was significantly reduced after eight days of antibiotic treatment (Fig. 2H). Next, we used PICRUST to predict changes in Kyoto Encyclopedia of Genes and Genomes (KEGG) pathways based on the results of 16S rRNA sequencing. The heatmap of level3 KEGG pathway abundances showed that antibiotic treatment significantly affected lipid and carbohydrate metabolism (Fig. 2I).

### The changes in gut microbes induced by non-absorbable antibiotic treatment do not affect the immune system

To identify the mechanism by which non-absorbable antibiotic treatment inhibited CRC metastasis, we first analyzed changes in the immune microenvironment in the liver. We observed a slight increase in CD8 T cells in the liver after non-absorbable antibiotic treatment, but no changes in other cells were observed (Fig. S2). As absorbed antibiotics have been reported to alter the concentrations of NK cell subtypes 22, we assessed changes in NK cell subtypes using antibodies against the markers CD11b and CD27. Non-absorbable antibiotic treatment had no effect on the proportion of CD11b+CD27+ NK cells, which play a role in anti-tumor immunity (Fig. S3A-B). Moreover, we analyzed the body's immune response to non-absorbable antibiotic treatment by evaluating immune cells in the spleen, colon and mesenteric lymph nodes. No meaningful changes in immune cells were detected after non-absorbable antibiotic treatment (Fig. S3C-3F).

### The changes in gut microbes induced by non-absorbable antibiotic treatment significantly affect bile acid metabolism

**Figure 3:**
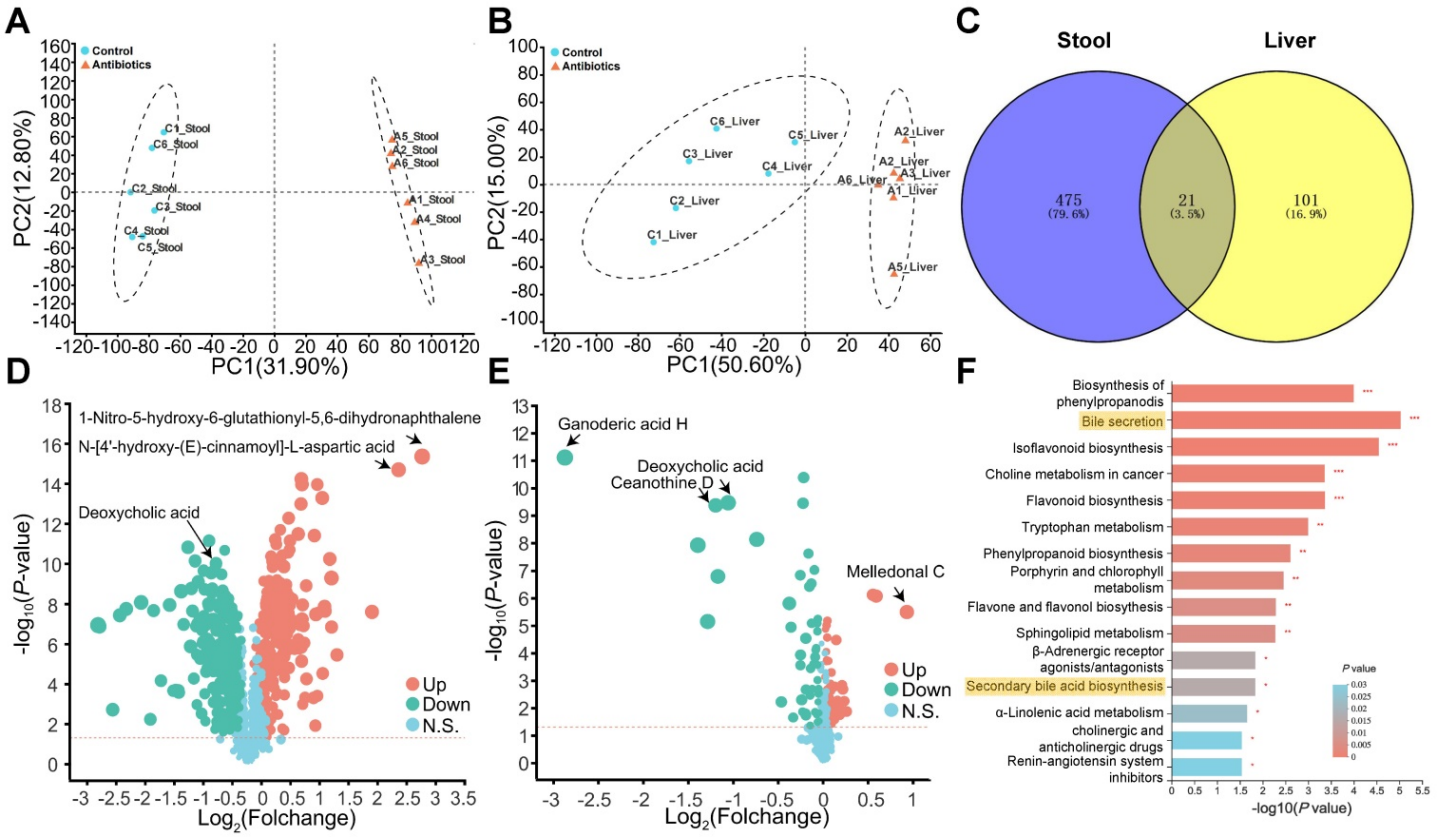
** Non-targeted metabolomics analysis reveals changes in bile acid metabolism. (A)** Fecal and **(B)** liver metabolites were altered after antibiotic treatment. **(C)** Venn plot of common differentially expressed metabolites between fecal and liver tissue samples. **(D)** Volcano plot of non-targeted metabolomics results in fecal samples and **(E)** liver tissues. **(F)** KEGG enrichment analysis of fecal metabolites showed that antibiotic treatment influenced the bile acid pathway. *P < 0.05, **P <0.01, *** P < 0.001.

We next evaluated changes in metabolism after antibiotic treatment. Fecal and hepatic metabolic profiles were investigated using LC-MS-based metabolomics. In total, 1022 fecal metabolites and 580 hepatic metabolites were detected, and PCA analysis indicated non-overlap between the control group and antibiotic treatment group (Fig. 3A and 3B). The lack of overlap suggests that the metabolites were mostly different between the two groups. Analysis of the metabolites using Student's t-test revealed 496 and 122 differently expressed metabolites (DEMs) in feces and the liver, respectively. A Venn plot of these DEMs showed that 21 were altered in both feces and the liver (Fig. 3C). In addition, a volcano plot revealed that antibiotic treatment significantly reduced DCA levels in feces and the liver (Fig. 3D and 3E). KEGG pathway enrichment analysis indicated changes in pathways related to bile acid secretion and secondary bile acid biosynthesis in feces, which were most likely due to changes in gut microbes (Fig. 3F).

### Targeted metabolomics shows that secondary bile acids are reduced after antibiotic treatment

**Figure 4:**
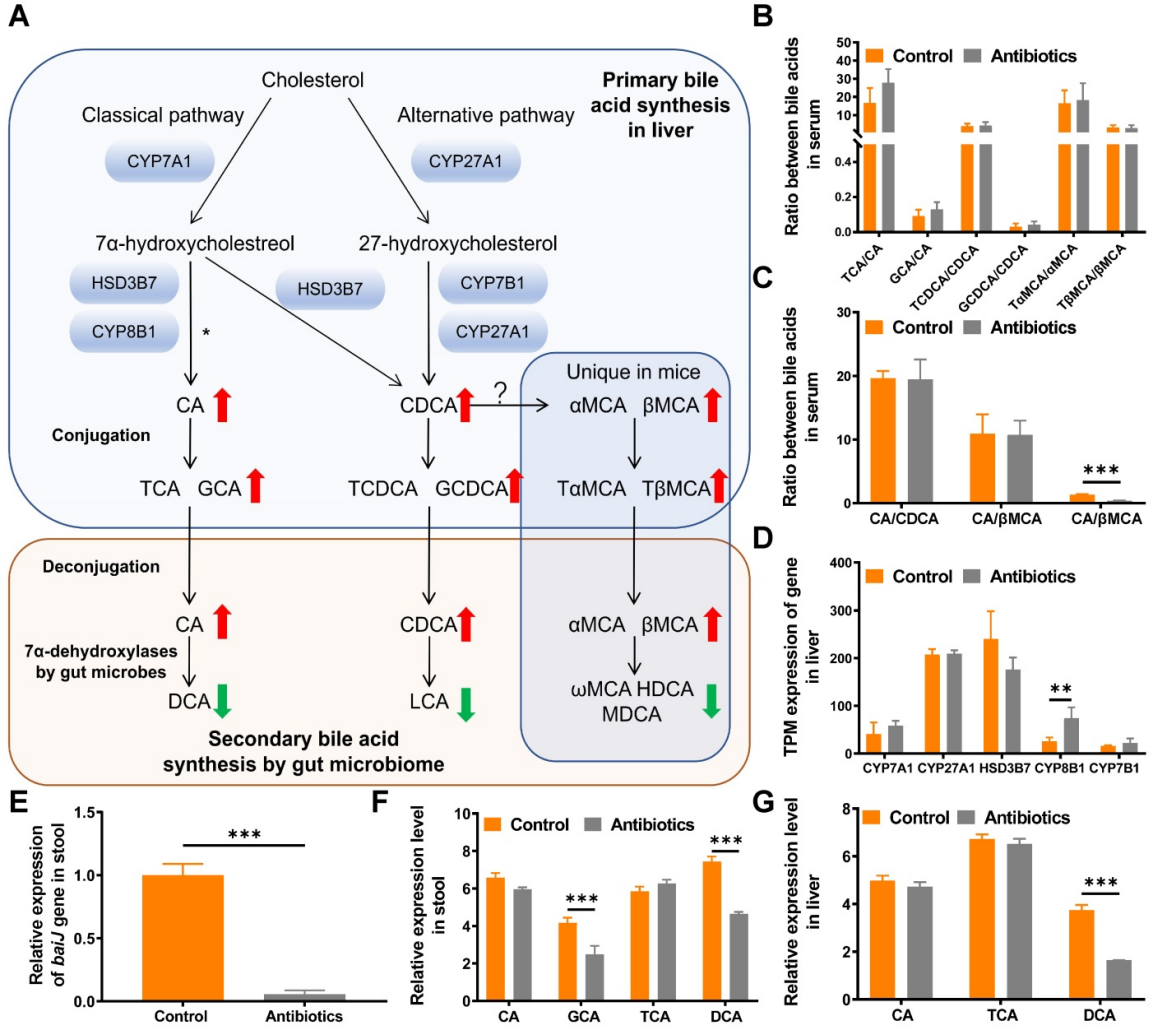
** Metabolomics analysis reveals that DCA is a key metabolite. (A)** Bile acid metabolism pathway. The red upward arrow indicates increasing levels of metabolites in serum after two weeks of treatment with non-absorbable antibiotics. The green downward arrow indicates decreasing levels of metabolites in serum after two weeks of treatment with non-absorbable antibiotics. **(B)** Ratio of conjugated primary bile acids to primary bile acids. **(C)** Ratios of primary bile acids. **(D)** Expression of bile acid synthesis-related genes in transcripts per million (TPM). **(E)** Quantitative PCR analysis of the *BaiJ* gene of *Clostridium* XIVa showed that antibiotic treatment significantly reduced its expression. **(F)** Relative expression of bile acid metabolites in mouse fecal samples and **(G)** liver tissues. *P < 0.05, ** *P* < 0.01, *** P < 0.001; CA, cholic acid; CDCA, chenodeoxycholic acid; αMCA, alpha-muricholic acid; βMCA, beta-muricholic acid; TCA, taurocholic acid; GCA, glycine cholic acid; TCDCA, taurochenodeoxycholic acid; GCDCA, glycochenodeoxycholic acid; TαMCA, tauro-α-muricholic acid; TβMCA, tauro-β-muricholic acid; DCA, deoxycholic acid; LCA, lithocholic acid; ωMCA, omega-muricholic acid; HDCA, hyodeoxycholic acid; MDCA, murideoxycholic acid.

Primary bile acids including CA, chenodeoxycholic acid (CDCA), α-muricholic acid (αMCA) and β-muricholic acid (βMCA) are produced via cholesterol clearance in the liver in mice. These bile acids are then conjugated with taurine or glycine and transported to the intestine to be metabolized by gut microbes (Fig. 4A). To systematically analyze changes in the bile acid pathway, targeted metabolomics of serum bile acids was performed. The results showed that primary bile acids were upregulated and secondary bile acids were downregulated after non-absorbable antibiotic treatment (Fig. 4A and 4B, Table S1). We then examined whether the use of non-absorbable antibiotics shifted bile acid biosynthesis from the classical pathway to the alternative pathway. The ratio of CA to βMCA was increased after antibiotic treatment, which might contribute to the feedback mechanism of bile acid synthesis (Fig. 4C). To verify that the alteration of secondary bile acid production was due to changes in gut microbes, we further analyzed the expression levels of genes related to primary bile acid synthesis. As expected, no significant changes were observed in the expression of genes encoding rate-limiting enzymes (CYP7A1, CYP27A1, HSD3B7, CYP7B1), thus demonstrating that the alteration of bile acids was due only to the decrease in gut microbe abundance (Fig. 4D).

### DCA is a key metabolite in metabolomics results

To substantiate the decrease in the relative abundance of DCA in feces and the liver, real-time PCR was used to quantify the expression of *BaiJ*, a gene involved in CA 7α-dehydroxylation by *Clostridium* XIVa, the only bacterial strain in the gut that can transform CA to DCA via 7α-dehydroxylation 17. As expected, the relative expression of *BaiJ* was remarkably reduced in the antibiotic treatment group (Fig. 4E).

We then analyzed all bile acid-related metabolites detected by metabolomics. Among all metabolites, DCA exhibited the greatest decrease in response to antibiotic treatment in fecal and liver samples, while no change in CA was observed (Fig. 4F and 4G). Moreover, in targeted metabolomics of bile acids, DCA exhibited the greatest change (Table S1). Thus, we decided to study the function of DCA in tumor proliferation and metastasis.

### DCA promotes cell proliferation and metastasis *in vitro*

**Figure 5:**
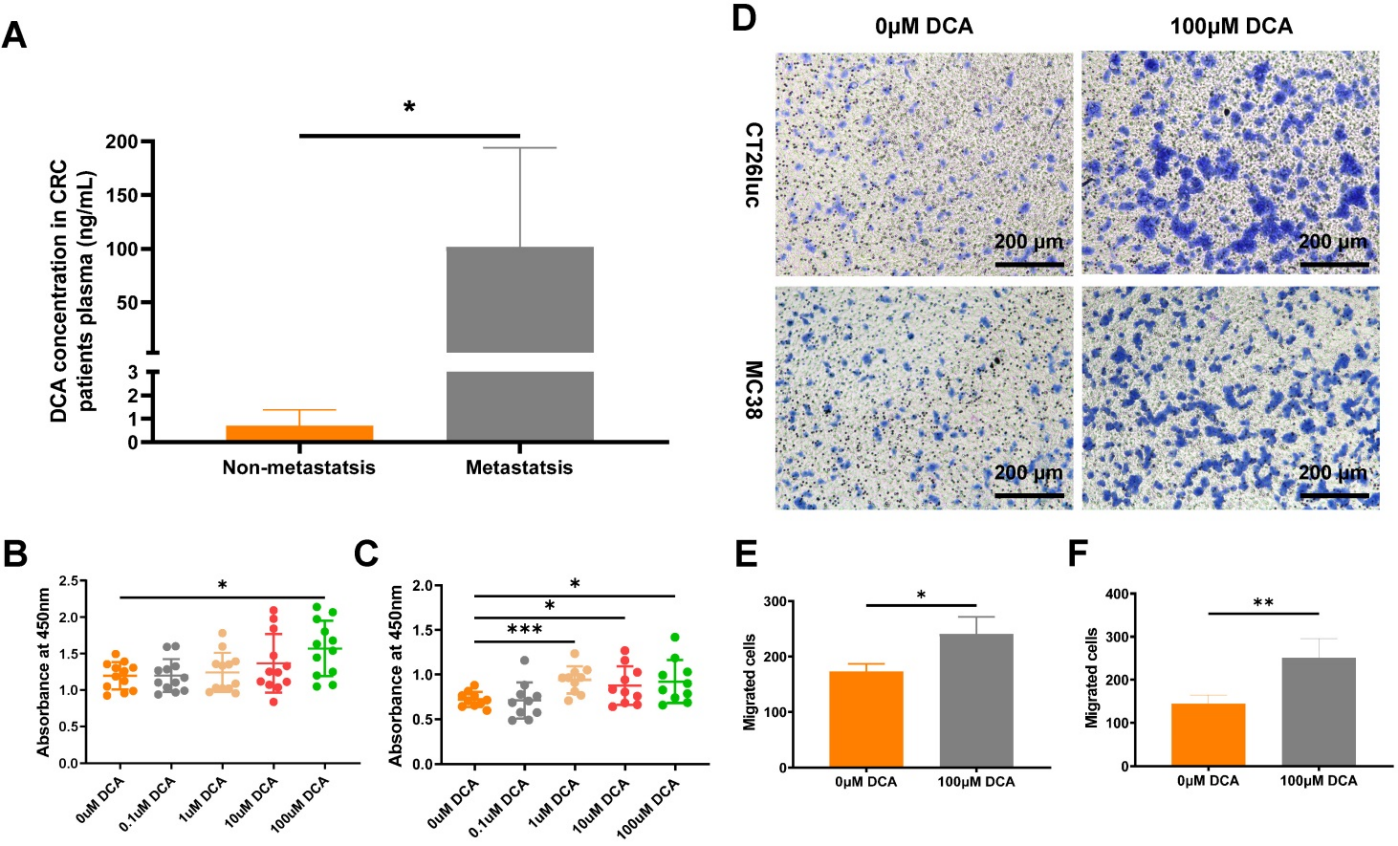
**DCA promotes cell proliferation and metastasis *in vitro*. (A)** DCA concentration in plasma of CRC patients. CCK8 assays of **(B)** CT26luc and **(C)** MC38 cells. **(D)** Transwell analysis showed that DCA promoted tumor cell migration. The results were observed under a microscope at 100×. Barplot of Transwell results of **(E)** CT26luc and **(F)** MC38 cells. *P < 0.05, **P < 0.01, *** P < 0.001.

Vahabi et al. found DCA concentrations in serum were higher in the high stage (stage Ⅱ, Ⅲ, Ⅳ) compared with the low stage (stage 0, Ⅰ) of CRC patients 23. To confirm the clinical DCA level in CRC patients, we analyzed the DCA concentration in plasma through LC-MS method. Result showed that plasma DCA concentrations in metastatic patients (n = 7) were significantly higher than in non-metastatic patients (n = 7, Fig. 5A).

To verify the effect of DCA on cancer cells, we used the CCK8 assay to examine the proliferation of CT26luc and MC38 cells in medium containing DCA. Compared with the control group, DCA promoted the proliferation of CT26luc and MC38 cells (Fig. 5B-C). As a DCA concentration of 100 μM had an obvious effect on cell proliferation, we used this concentration in Transwell experiments, which showed that DCA could enhance the migration of cancer cells (Fig. 5D-F).

### DCA promotes tumor metastasis *in vivo*

**Figure 6:**
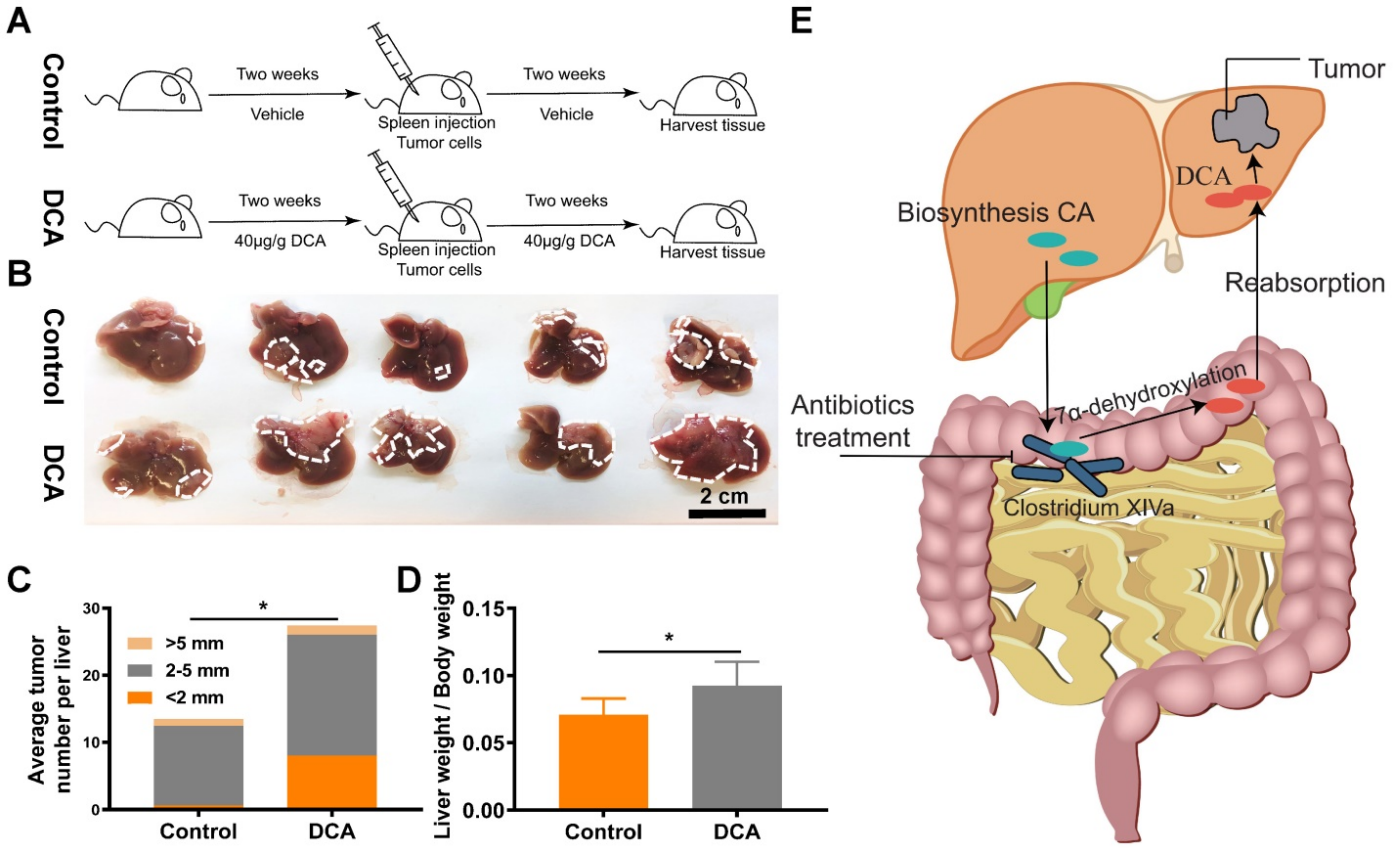
** DCA promotes cell proliferation and metastasis *in vivo*. (A)** Flowchart of mouse treatment with DCA. **(B)** DCA treatment promoted colorectal cancer liver metastasis in the mouse model. Analysis of **(C)** the average number of tumors per liver and **(D)** metastasis indexes showed that DCA promoted liver metastasis *in vivo*. **(E)** Schematic illustration of the experimental results. *P < 0.05.

Next, we verified the effects of DCA on a mouse model of hepatic metastasis established by spleen injection of MC38 cells (Fig. 6A). Since DCA was anticipated to facilitate metastasis in the mouse model, fewer cells (1.5×10^6^ MC38 cells) than normal (2×10^6^ MC38 cells) were used to construct the hepatic metastasis model. As expected, DCA treatment promoted liver metastasis of CRC *in vivo* (Fig. 6B). The average number of tumors per liver and the metastasis index were increased in the mouse model after treatment with DCA (Fig. 6C and 6D). Overall, we found that non-absorbable antibiotic treatment significantly decreased the abundance of Clostridium XIVa and decreased the concentration of DCA, a molecule capable of promoting tumor proliferation and metastasis (Fig. 6E).

## Discussion

The colon and rectum are common sites of tumor formation and feature a complex system of host cells and microbes 24. In industrialized countries, CRC and CRC-related adenomas affect a quarter of the population 25, 26. The cure rate for localized disease ranges from 70% to 90%, but the mortality rate for advanced CRC is high, making CRC the third leading cause of cancer mortality worldwide 2, 27. The occurrence and development of CRC have been linked to genetic mutations and environmental effects 28, 29. Microbes, which are present as a large population in the intestinal tract and function in symbiosis with the body of the host, have been reported to play an important role in CRC occurrence 30-32. However, the potential influence of microbes on CRC metastasis has been poorly studied 14. Treatment with ABX is often used to alter the microbial composition of the gut; however, these cocktails contain metronidazole and ampicillin, and the systemic bioavailability of these antibiotics from the gut may have potential effects on distal organs 14. Therefore, to probe potential links with CRC metastasis, it is important to directly target gut microbes. In this study, we constructed mouse models for treatment with the non-absorbable antibiotics gentamicin and amikacin, which have minimal bioavailability from the intestine 33, 34. The subsequent systematic analysis of changes in immune and metabolite composition demonstrated that intestinal microbes promote tumor metastasis by modulating DCA metabolism.

Some microbial species have been shown to have direct effects on carcinogenesis and tumor development. Pks+ *Escherichia coli*, *Fusobacterium nucleatum*, and enterotoxigenic *Bacteroides fragilis* could promote the incidence and development of CRC by direct toxic effects on DNA to induce mutations or increase carcinoma cell proliferation 8. In addition, orally derived *Fusobacterium nucleatum* has been found to play a potential role in the metastasis of breast cancer and CRC. This bacterium could colonize to the breast and colon in a Fap2-Gal-GalNAc-dependent manner and promote breast metastasis by inhibiting T cell aggregation or promote CRC metastasis by the KRT7-AS/KRT7 pathway 35, 36. Our study aimed to identify specific bacteria that promote resistance to CRC metastasis. Our analysis of differential microbial abundance at the species level based on 16S rRNA sequencing demonstrated that *Parabacteroides goldsteinii*, *Bacteroides intestinalis* and *Bacteroides uniformis* were significantly upregulated in the antibiotic treatment group. These bacteria have been previously associated with glucose and lipid metabolism in humans as well as obesity 37, 38. It is increasingly evident that adipose tissue supports the survival of metastatic cancer cells, allowing tumors to form at distant sites 39. Adipose tissue can promote cancer metastasis via multiple routes, including secretion of soluble adipokines and inflammatory factors, promotion of angiogenic processes, changes in metabolism in cancer cells, and alteration of the extracellular matrix 39, 40. In the present study, no significant effect of antibiotic treatment on the body weight of mice was observed over the two-week treatment period. The roles of these bacteria in glucose and lipid metabolism and their relationship with obesity and cancer require further study.

Immune cells are closely related to cancer metastasis. In addition to cytotoxic T lymphocytes and NK cells, which are involved in direct killing of tumor cells, infiltrating leukocytes can regulate angiogenesis, tissue remodeling and pre-metastasis niches to influence the metastasis of neoplastic cells 41. Commensals can modulate the development and function of various immune cell populations to regulate the primary and adaptive immunity of the body through immunostimulatory or immunomodulatory effects, highlighting the importance of these microbes in the process of cancer development and metastasis 42, 43. To explore the potential mechanisms of inhibition of metastasis, we analyzed changes in the immune system in the antibiotic treatment group. However, no significant changes in the body immune state and the microenvironment of liver immune cells were observed. Only CD8 T cells in the liver increased slightly after non-absorbable antibiotic treatment, thus indicating that antibiotic treatment likely affects tumor metastasis in our mouse model via non-immune-related mechanisms.

Next, we examined changes in metabolism, as intestinal microbes can affect immune cell function and regulate cell proliferation and death via regulation of host metabolism. In particular, short-chain fatty acids and bile acids produced by microbes have been shown to be critical in the occurrence and development of cancer. According to numerous experimental studies, short-chain fatty acids protect against the occurrence and development of CRC, help maintain colon movement, improve visceral blood flow, reduce inflammation and inhibit the proliferation and migration of tumor cells 44-46. By contrast, bile acids produced in fat may promote the occurrence and development of cancer 47, 48. Recent studies have confirmed the cancer-promoting mechanism of bile acids in primary CRC 49, 50. In mice, a high-fat diet or genetic susceptibility and obesity promoted CRC development by increasing bile acid levels, which led to intestinal barrier impairment and enhanced IL-6/STAT3-related inflammation 51. In the present study, using non-targeted and targeted metabolomics, we found that antibiotic treatment increased primary bile acids and decreased secondary bile acids in serum, and these alterations were due solely to changes in intestinal microbes. The combined results of fecal and liver non-targeted metabolomics indicated that DCA was the bile acid metabolite most impacted by antibiotic treatment. DCA is a secondary bile acid formed by the metabolism of the primary bile acid CA by intestinal microbes and has been implicated in CRC and liver cancer development 17, 52, 53. In human cancer cells, DCA has been found to promote proliferation and migration *in vitro* through activation of the β-catenin or protein kinase C pathway 53, 54. We confirmed that the changes in DCA were the result of reduced microbial diversity and further verified the ability of DCA to promote tumor proliferation and metastasis both *in vitro* and *in vivo*.

Taken together, our results indicate that non-absorbable antibiotic treatment significantly affect gut microbes and decrease DCA concentrations in the body, thereby reducing CRC proliferation and metastasis. This work revealed that DCA may have clinical potential as a biomarker or therapeutic target for reducing CRC metastasis.

## Supplementary Material

Supplementary figures and table.

## Abbreviations

ABX: antibiotic cocktail
ALT: alanine aminotransferase
CA: cholic acid
CRC: colorectal cancer
DCA: deoxycholic acid
LC-MS: liquid chromatography-mass spectrometry
PCA: principal component analysis
OUT: operational taxonomic unit
QPCR: quantitative PCR
SPF: specific pathogen-free

## References

[1] Araghi M, Soerjomataram I, Jenkins M, Brierley J, Morris E, Bray F (2019). Global trends in colorectal cancer mortality: projections to the year 2035. International journal of cancer.

[2] Meyerhardt JA, Mayer RJ (2005). Systemic therapy for colorectal cancer. N Engl J Med.

[3] Zhang Q, Zhang H, Ding J, Liu H, Li H, Li H (2018). Combination Therapy with EpCAM-CAR-NK-92 Cells and Regorafenib against Human Colorectal Cancer Models. J Immunol Res.

[4] August DA, Ottow RT, Sugarbaker PH (1984). Clinical perspective of human colorectal cancer metastasis. Cancer Metastasis Rev.

[5] Bissell MJ, Hines WC (2011). Why don't we get more cancer? A proposed role of the microenvironment in restraining cancer progression. Nature medicine.

[6] Dzutsev A, Badger JH, Perez-Chanona E, Roy S, Salcedo R, Smith CK (2017). Microbes and Cancer. Annu Rev Immunol.

[7] Kaiser J (2017). Gut microbes shape response to cancer immunotherapy. Science.

[8] Sears CL, Garrett WS (2014). Microbes, microbiota, and colon cancer. Cell Host Microbe.

[9] Li T, Apte U (2015). Bile Acid Metabolism and Signaling in Cholestasis, Inflammation, and Cancer. Adv Pharmacol.

[10] Gérard P (2014). Metabolism of cholesterol and bile acids by the gut microbiota. Pathogens.

[11] Nguyen TT, Ung TT, Kim NH, Jung YD (2018). Role of bile acids in colon carcinogenesis. World J Clin Cases.

[12] Dong W, Liu L, Dou Y, Xu M, Liu T, Wang S (2018). Deoxycholic acid activates epidermal growth factor receptor and promotes intestinal carcinogenesis by ADAM 17-dependent ligand release. Journal of cellular and molecular medicine.

[13] Liu L, Dong W, Wang S, Zhang Y, Liu T, Xie R (2018). Deoxycholic acid disrupts the intestinal mucosal barrier and promotes intestinal tumorigenesis. Food Funct.

[14] Rosean CB, Bostic RR, Ferey JC, Feng T-Y, Azar FN, Tung KS (2019). Preexisting commensal dysbiosis is a host-intrinsic regulator of tissue inflammation and tumor cell dissemination in hormone receptor-positive breast cancer. Cancer research.

[15] Ma C, Han M, Heinrich B, Fu Q, Zhang Q, Sandhu M (2018). Gut microbiome-mediated bile acid metabolism regulates liver cancer via NKT cells. Science.

[16] Xu J, Xu H-m, Peng Y, Zhao C, Zhao H-l, Huang W (2021). The effect of different combinations of antibiotic cocktails on mice and selection of animal models for further microbiota research. Applied Microbiology and Biotechnology.

[17] Yoshimoto S, Loo TM, Atarashi K, Kanda H, Sato S, Oyadomari S (2013). Obesity-induced gut microbial metabolite promotes liver cancer through senescence secretome. Nature.

[18] Wei X, Zhang J, Gu Q, Huang M, Zhang W, Guo J (2017). Reciprocal expression of IL-35 and IL-10 defines two distinct effector Treg subsets that are required for maintenance of immune tolerance. Cell reports.

[19] Langille MG, Zaneveld J, Caporaso JG, McDonald D, Knights D, Reyes JA (2013). Predictive functional profiling of microbial communities using 16S rRNA marker gene sequences. Nature biotechnology.

[20] Wickham H (2011). ggplot2. Wiley Interdisciplinary Reviews: Computational Statistics.

[21] Sethi V, Kurtom S, Tarique M, Lavania S, Malchiodi Z, Hellmund L (2018). Gut Microbiota Promotes Tumor Growth in Mice by Modulating Immune Response. Gastroenterology.

[22] D'Alessandro G, Antonangeli F, Marrocco F, Porzia A, Lauro C, Santoni A (2020). Gut microbiota alterations affect glioma growth and innate immune cells involved in tumor immunosurveillance in mice. Eur J Immunol.

[23] Vahabi F, Sadeghi S, Arjmand M, Mirkhani F, Hosseini E, Mehrabanfar M (2017). Staging of colorectal cancer using serum metabolomics with 1HNMR Spectroscopy. Iranian journal of basic medical sciences.

[24] Siegel RL, Miller KD, Goding Sauer A, Fedewa SA, Butterly LF, Anderson JC (2020). Colorectal cancer statistics, 2020. CA Cancer J Clin.

[25] Xie YH, Chen YX, Fang JY (2020). Comprehensive review of targeted therapy for colorectal cancer. Signal Transduct Target Ther.

[26] Wong SH, Yu J (2019). Gut microbiota in colorectal cancer: mechanisms of action and clinical applications. Nat Rev Gastroenterol Hepatol.

[27] Marmol I, Sanchez-de-Diego C, Pradilla Dieste A, Cerrada E, Rodriguez Yoldi MJ (2017). Colorectal Carcinoma: A General Overview and Future Perspectives in Colorectal Cancer. Int J Mol Sci.

[28] Si H, Yang Q, Hu H, Ding C, Wang H, Lin X (2021). Colorectal cancer occurrence and treatment based on changes in intestinal flora. Semin Cancer Biol.

[29] Markowitz SD, Bertagnolli MM (2009). Molecular origins of cancer: Molecular basis of colorectal cancer. N Engl J Med.

[30] Kostic AD, Chun E, Meyerson M, Garrett WS (2013). Microbes and inflammation in colorectal cancer. Cancer Immunol Res.

[31] Uronis J, Jobin C (2009). Microbes and colorectal cancer: is there a relationship?. Current oncology.

[32] Van Raay T, Allen-Vercoe E (2017). Microbial Interactions and Interventions in Colorectal Cancer. Microbiol Spectr.

[33] Hu Z, Tawa R, Konishi T, Shibata N, Takada K (2001). A novel emulsifier, labrasol, enhances gastrointestinal absorption of gentamicin. Life Sci.

[34] Amir M, Ansari VA, Sirbaiya AK, Zishan M (2017). ENHANCEMENT OF ORAL UPTAKE OF AMIKACIN USING COPOLYMERS. Journal of Drug Delivery and Therapeutics.

[35] Chen S, Su T, Zhang Y, Lee A, He J, Ge Q (2020). Fusobacterium nucleatum promotes colorectal cancer metastasis by modulating KRT7-AS/KRT7. Gut Microbes.

[36] Parhi L, Alon-Maimon T, Sol A, Nejman D, Shhadeh A, Fainsod-Levi T (2020). Breast cancer colonization by Fusobacterium nucleatum accelerates tumor growth and metastatic progression. Nat Commun.

[37] Wu TR, Lin CS, Chang CJ, Lin TL, Martel J, Ko YF (2019). Gut commensal Parabacteroides goldsteinii plays a predominant role in the anti-obesity effects of polysaccharides isolated from Hirsutella sinensis. Gut.

[38] Lopez-Almela I, Romani-Perez M, Bullich-Vilarrubias C, Benitez-Paez A, Gomez Del Pulgar EM, Frances R (2021). Bacteroides uniformis combined with fiber amplifies metabolic and immune benefits in obese mice. Gut Microbes.

[39] Annett S, Moore G, Robson T (2020). Obesity and Cancer Metastasis: Molecular and Translational Perspectives. Cancers (Basel).

[40] Wishart AL, Conner SJ, Guarin JR, Fatherree JP, Peng Y, McGinn RA (2020). Decellularized extracellular matrix scaffolds identify full-length collagen VI as a driver of breast cancer cell invasion in obesity and metastasis. Science advances.

[41] DeNardo DG, Johansson M, Coussens LM (2008). Immune cells as mediators of solid tumor metastasis. Cancer and Metastasis Reviews.

[42] Ivanov II, Honda K (2012). Intestinal commensal microbes as immune modulators. Cell host & microbe.

[43] Honda K, Littman DR (2012). The microbiome in infectious disease and inflammation. Annu Rev Immunol.

[44] Frank DN, Pace NR (2008). Gastrointestinal microbiology enters the metagenomics era. Curr Opin Gastroenterol.

[45] Salonen A, Lahti L, Salojarvi J, Holtrop G, Korpela K, Duncan SH (2014). Impact of diet and individual variation on intestinal microbiota composition and fermentation products in obese men. ISME J.

[46] Hinnebusch BF, Meng S, Wu JT, Archer SY, Hodin RA (2002). The effects of short-chain fatty acids on human colon cancer cell phenotype are associated with histone hyperacetylation. J Nutr.

[47] Greenwald P, Clifford CK, Milner JA (2001). Diet and cancer prevention. Eur J Cancer.

[48] Grosso G, Bella F, Godos J, Sciacca S, Del Rio D, Ray S (2017). Possible role of diet in cancer: systematic review and multiple meta-analyses of dietary patterns, lifestyle factors, and cancer risk. Nutr Rev.

[49] Ocvirk S, O'Keefe SJ (2017). Influence of Bile Acids on Colorectal Cancer Risk: Potential Mechanisms Mediated by Diet - Gut Microbiota Interactions. Curr Nutr Rep.

[50] Katsidzira L, Ocvirk S, Wilson A, Li J, Mahachi C, Soni D (2019). Differences in fecal gut microbiota, short-chain fatty acids and bile acids link colorectal cancer risk to dietary changes associated with urbanization among zimbabweans. Nutrition and cancer.

[51] Wang S, Dong W, Liu L, Xu M, Wang Y, Liu T (2019). Interplay between bile acids and the gut microbiota promotes intestinal carcinogenesis. Molecular carcinogenesis.

[52] Bayerdorffer E, Mannes GA, Richter WO, Ochsenkuhn T, Wiebecke B, Kopcke W (1993). Increased serum deoxycholic acid levels in men with colorectal adenomas. Gastroenterology.

[53] Pai R, Tarnawski AS, Tran T (2004). Deoxycholic acid activates beta-catenin signaling pathway and increases colon cell cancer growth and invasiveness. Mol Biol Cell.

[54] Lin R, Zhan M, Yang L, Wang H, Shen H, Huang S (2020). Deoxycholic acid modulates the progression of gallbladder cancer through N 6-methyladenosine-dependent microRNA maturation. Oncogene.

