# Supplementary material for: Non-absorbable antibiotic treatment inhibits colorectal cancer liver metastasis by modulating deoxycholic acid metabolism by intestinal microbes
Source: J Cancer. 2022 Jan 1;13(3):764–74. doi: 10.7150/jca.63490 (PMC8824892; doi:10.7150/jca.63490)
Supplement: Supplementary file 1 — Supplementary figures and table. [file jcav13p0764s1.pdf]

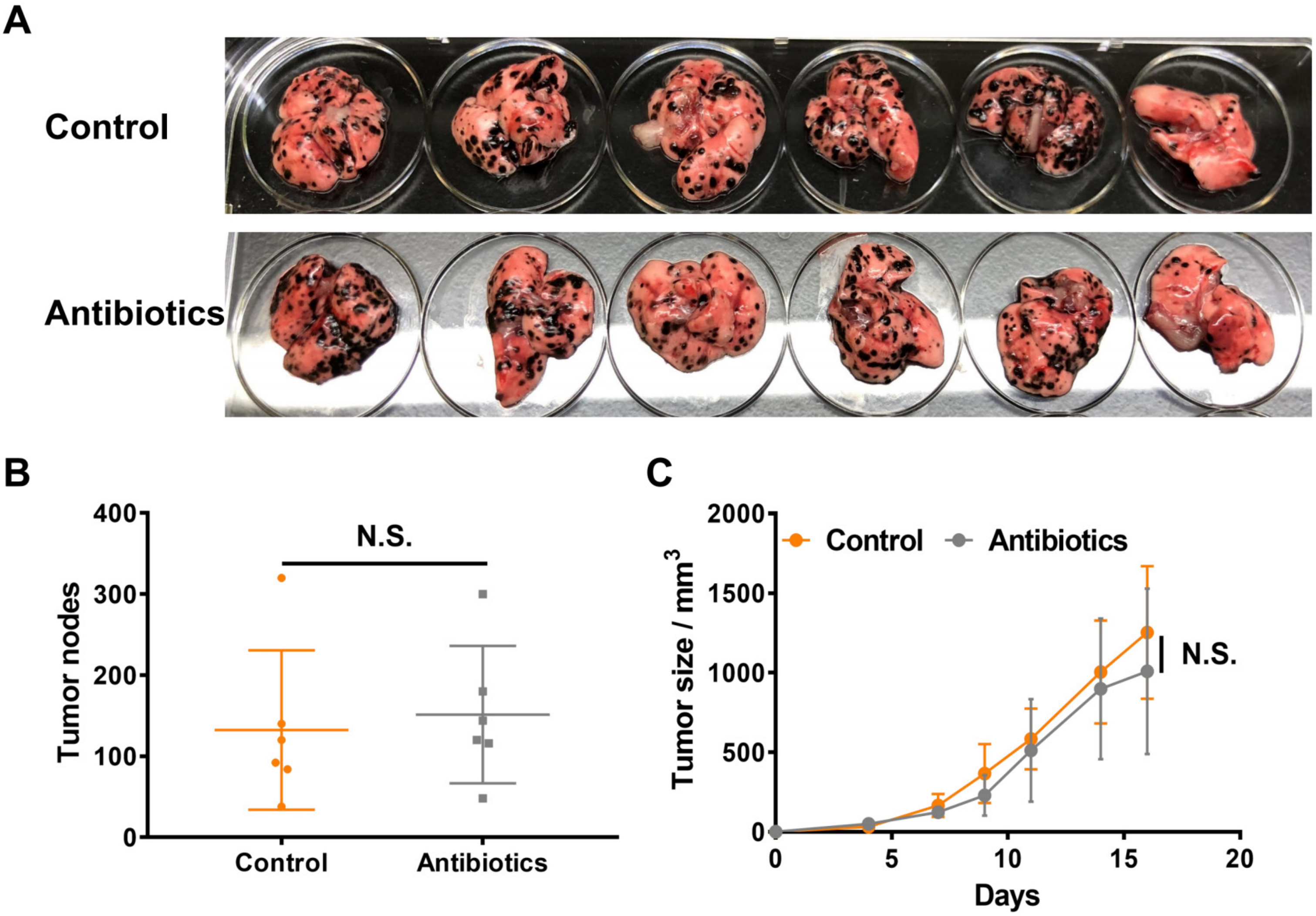

Supplementary materials

Supplementary Figure 1. Non-absorbable antibiotic treatment does not influence tumor lung metastasis and subcutaneous flank tumor proliferation in a mouse model. (A) No significant difference was observed between the control group and the antibiotic treatment group in a tail vein injection lung metastasis model in C57BL/6 mice. (B) Number of metastatic tumor nodes in the lungs. (C) Curve of tumor size in the subcutaneous flank tumor proliferation model. N.S., no significance.

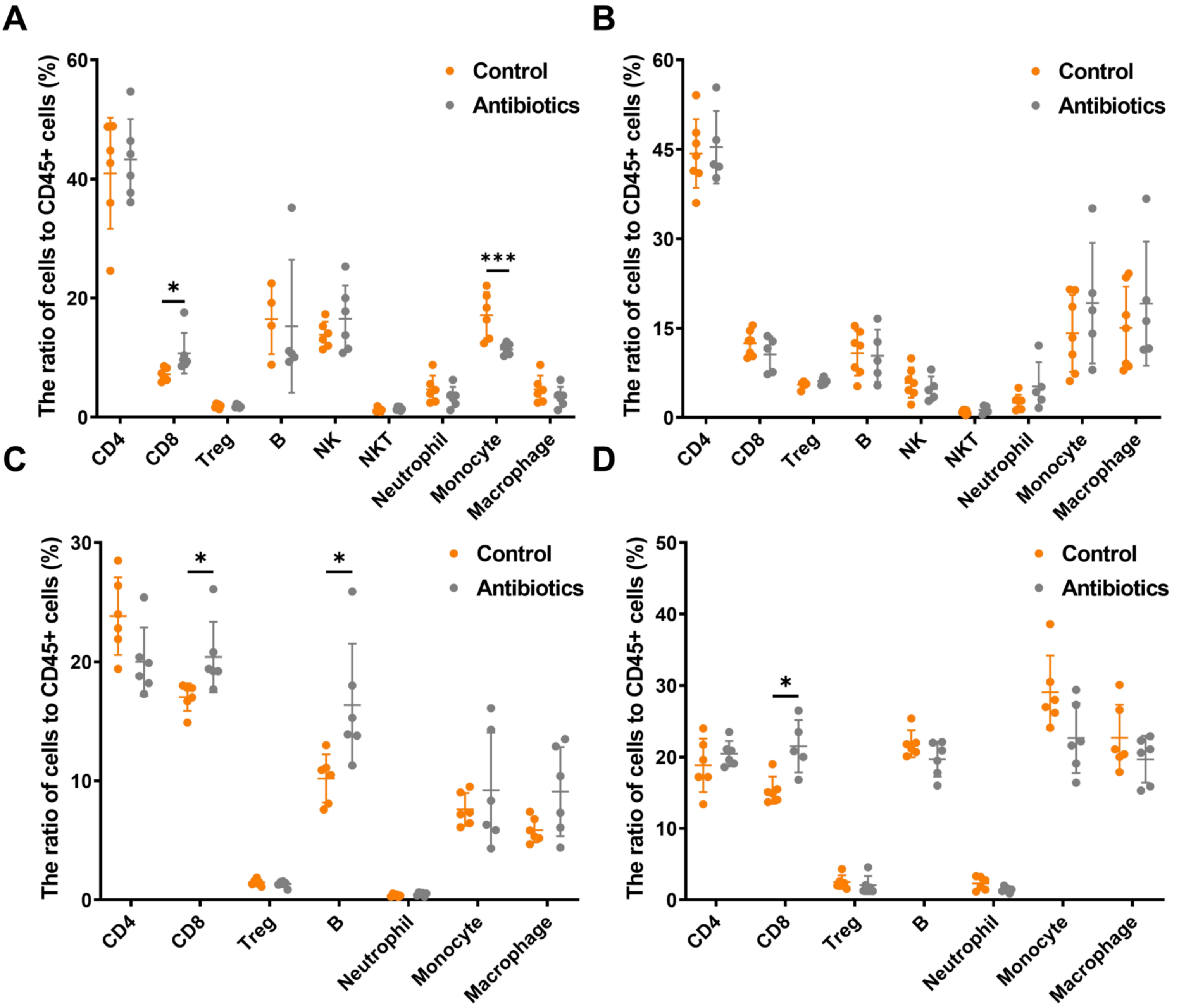

Supplementary Figure 2. Non-absorbable antibiotic treatment slightly increases the proportion of CD8 T cells the in liver. (A) Changes in liver immune cells in BALB/c mice without tumors after treatment with non-absorbable antibiotics for two weeks and (B) in BALB/c mice with metastatic tumors after treatment with non-absorbable antibiotics for four weeks. (C) Changes in liver immune cells in C57BL/6J mice without tumors after treatment with non-absorbable antibiotics for two weeks and (D) in C57BL/6J mice with metastatic tumors after treatment with non-absorbable antibiotics for four weeks. *$P < 0.05$, ***$P < 0.001$.

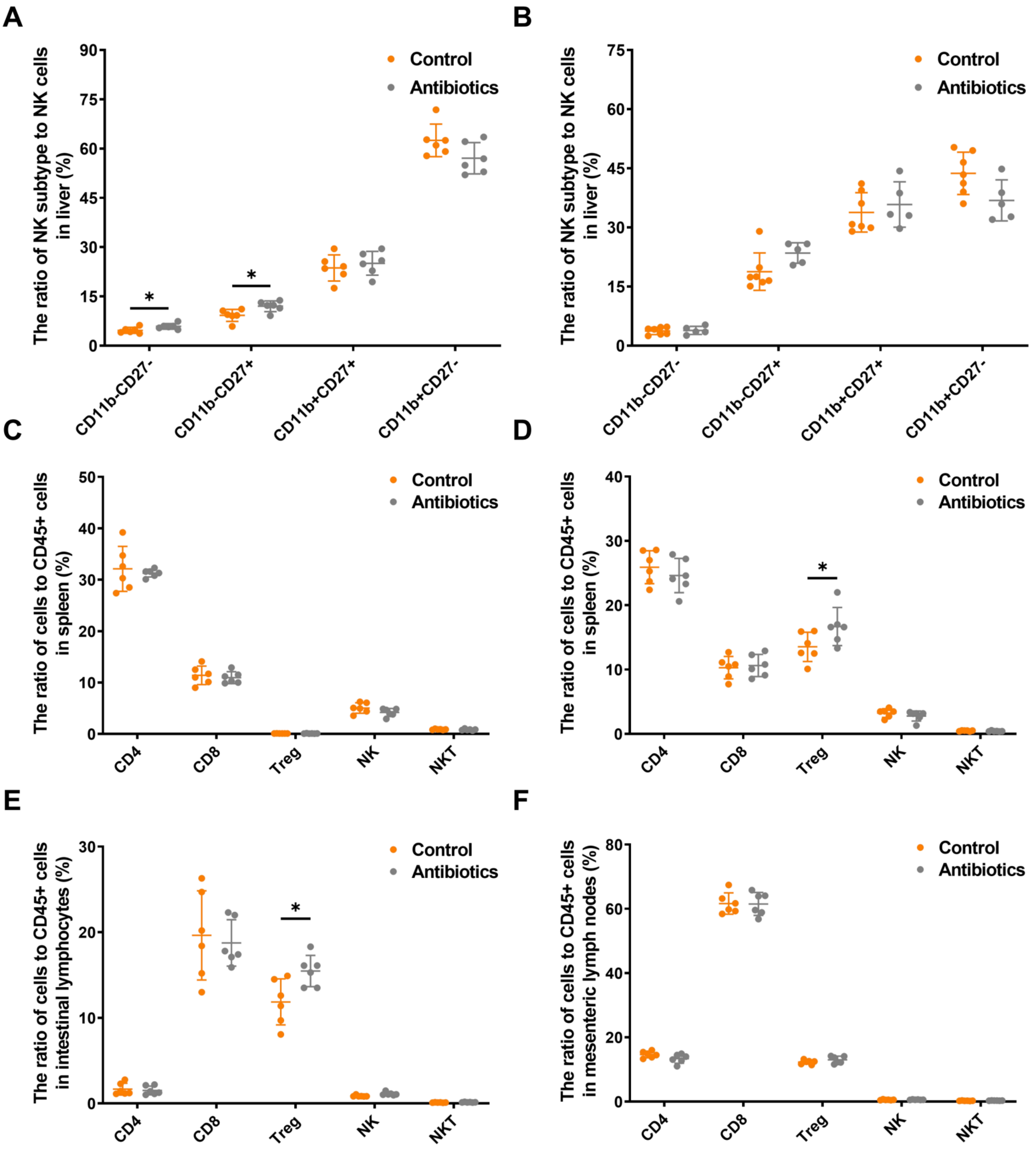

Supplementary Figure 3. Non-absorbable antibiotic treatment has no obvious effect

on the immune system. (A) Changes in NK cell subtypes in BALB/c mice without tumors after treatment with non-absorbable antibiotics for two weeks and (B) in BALB/c mice with metastatic tumors after treatment with non-absorbable antibiotics for four weeks. (C) Spleen infiltrating immune cells in BALB/c mice without tumors after treatment with non-absorbable antibiotics for two weeks and (D) in BALB/c mice with metastatic tumors after treatment with non-absorbable antibiotics for four weeks. (E) Colon and (F) mesenteric lymph node infiltrating immune cells in BALB/c mice after treatment with non-absorbable antibiotics for two weeks. *$P < 0.05$.

Subtype	Name	C1	C2	C3	A1	A2	A3	RSD
**Secondary bile acids**	Deoxycholic acid	217.60	145.15	119.95	8.90	22.05	21.65	10%
	Omega-Murichoclic acid	66.25	62.35	58.95	14.80	40.80	23.55	3%
	murideoxycholic acid	0.95	2.30	0.50	0.05	0.50	1.15	5%
	Hyodeoxycholic acid	11.75	11.25	9.90	3.60	5.80	6.60	8%
	Lithocholic acid	6.10	7.45	5.45	3.65	5.85	7.10	12%
**Primary bile acids**	Taurochenodeoxycholic acid	11.65	11.45	4.50	14.95	8.95	19.55	5%
	Tauro-α-muricholic acid	72.85	117.25	30.65	115.30	62.50	177.30	6%
	Alpha-Muricholic acid	5.45	4.75	2.75	4.15	6.90	10.10	6%
	Chenodeoxycholic acid	2.60	2.30	1.90	2.60	4.45	4.10	13%
	Cholic acid	48.30	44.50	39.70	41.50	91.40	89.85	8%
	Glycocholic acid	5.05	5.40	2.10	7.25	8.40	11.00	6%
	Glycochenodeoxycholic acid	0.05	0.05	0.10	0.15	0.10	0.20	9%
	Taurocholic acid	953.75	1019.75	297.45	1271.15	1746.25	3009.85	2%
	Tauro-β-muricholic acid	120.70	140.15	42.45	429.15	342.80	612.90	6%
	Beta-Muricholic acid	34.60	33.25	31.60	93.65	297.25	250.30	8%

(yellow)	**Standard for classical pathway of bile acid metabolism**	**A:antibiotic treatment group**
(green)	**Standard for alternative pathway of bile acid metabolism**	**C:control group**
(red)	**Standard for unique pathway in mice of bile acid metabolism**	

Supplementary Table 1. Serum bile acid concentrations in targeted metabolomics of bile acids.
